# Long noncoding RNA FAM3D-AS1 inhibits development of colorectal cancer through NF-κB signaling pathway

**DOI:** 10.1042/BSR20190724

**Published:** 2019-07-05

**Authors:** Ying Meng, Feng Yu

**Affiliations:** 1 The First Division of Cancer Department of Zibo Central Hospital, Shandong, China; 2 The Second Division of Gastroenterology Department of Zibo Central Hospital, Shandong, China

**Keywords:** Colorectal cancer, EMT, lncRNA

## Abstract

Although numerous differential long noncoding RNAs (IncRNAs) have been identified, the relationship between lncRNA and colorectal cancer (CRC) still remains largely unclear. In the present study, we investigated the function and provided a possible mechanism of lncRNA FAM3D-AS1 in CRC. CCK8, transwell, and trypan blue staining were used to evaluate the proliferation, invasion, and cell death rates. Real-time PCR was used to elucidate the expression level of FAM3D-AS1. We found that lncRNA FAM3D-AS1 located in cytoplasm. Overexpression of FAM3D-AS1 significantly inhibited the cell proliferation, cell survival rates, and invaded cells. We also provided evidence that FAM3D-AS1 reversed the EMT process. Moreover, we proved that FAM3D-AS1 inhibits CRC development through NF-κB signaling pathway. Taken together, we performed functional analysis of FAM3D-AS1 and provided a possible mechanism in the development of CRC. Our study provided new targets for clinical treatment.

## Introduction

Colorectal cancer (CRC) is the first most common cancer worldwide and the third leading cause in tumor [1]. Although numerous studies have been implemented in the treatment of CRC, such as surgical excision and adjuvant chemoradiotherapy [2], the long-term prognosis of patients remains disadvantageous [3,4]. Thus, exploring more and new biomarker and treatment are distinctly warranted.

Long noncoding RNA (lncRNA) is a specific noncoding RNA that longer than 200 nts and cannot code protein [5]. LncRNA has been widely studied in many diseases, such as heart development [6], tumor development [7], obesity [8], and kidney disease [9]. The function of lncRNA is also diverse, such as cell differenation [10], cell apoptosis [11], invasion [12], and cell cycle [13]. LncRNA AFAP1-AS1 plays an oncogenic role in promoting cell migration in nonsmall cell lung cancer [14]. LncRNA-MEG3 inhibits activation of hepatic stellate cells through SMO protein and miR-212 [15]. The mechanism of lncRNA mainly discussed about competing endogenous RNA [16], cis and trans mechanism [17] and influence transcription [18]. There is no doubt that exploring more and more lncRNAs will provide new insights for clinical treatment of CRC.

Although numerous differential lncRNAs have been identified, the relationship between lncRNA and CRC still remains largely unclear. In the present study, we investigated the function and provided a possible mechanism of lncRNA FAM3D-AS1 in CRC. We found that lncRNA FAM3D-AS1 located in cytoplasm. Overexpression of FAM3D-AS1 significantly inhibited the cell proliferation, cell survival rates, and invaded cells. We also provided evidence that FAM3D-AS1 reversed the EMT process. Moreover, we proved that FAM3D-AS1 inhibits CRC development through NF-κB signaling pathway. Taken together, we performed functional analysis of FAM3D-AS1 and provided a possible mechanism in the development of CRC. Our study provided new targets for clinical treatment.

## Materials and methods

### Cell culture and transfection

The cell lines, HCT-116, SW116, SW480, SW620, and HT29, used in the present study were purchased from American Type Culture Collection. HCT-116 was cultured in Ham’s F12K medium (Thermo Fisher Scientific, U.S.A.) containing 10% FBS (Life Technologies, Grand Island, U.S.A.). SW480 and SW620 were cultured in Leibovitz’s L-15 medium (Thermo Fisher Scientific, U.S.A.) containing 10% FBS (Life Technologies, GrandIsland, U.S.A.). SW116 and HT-29 cells were cultured in RPMI-1640 medium (Thermo Fisher Scientific, U.S.A.) containing 10% FBS (Life Technologies, Grand Island, U.S.A.). Cells were maintained at 37°C in a humidified atmosphere with 5% CO_2_.

### Plasmid construction and transfection

The FAM3D-AS1 overexpression vector was constructed by GenePharma (Shanghai, China). For the overexpression experiments, the transfection efficiency was verified in HCT116 cells through quantitative real-time PCR (qRT-PCR). We used Lipofectamine 2000 (Invitrogen, Carlsbad, CA, U.S.A.) to perform transfection according to the manufacturer’s protocol.

### Real-time PCR

Amplification of cDNA was performed with SYBR Green Real-time qPCR SuperMix UDG reagents (Invitrogen, Carlsbad, CA, U.S.A.) and the MX3000P system (Stratagene, La Jolla, CA. U.S.A.). The following cycling conditions were used: 95°C for 15 s, then 40 cycles of 95°C for 15 s, 60°C for 15 s, and 68°C for 20 s. The fold-change of lncRNA FAM3D-AS1 expression was calculated by the 2^−ΔΔ*C*^_T_ method following normalization to GAPDH.

### Western blotting

Proteins from HCT116 cells were extracted by using RIPA buffer. BCA assay was utilized to measure protein concentration. The extracted proteins were subjected to 10% SDS/PAGE and transferred onto 0.22 µm PVDF membranes (REF 03010040; Roche, Pleasanton, CA, U.S.A.). The membranes were blocked with 5% nonfat milk and incubated with anti-β-catenin, anti-E-cadherin, anti-N-cadherin, anti-Vimentin, anti-p65, anti-IκBα, anti-histone H1, anti-tubulin (all from Santa Cruz Biotechnology, Santa Cruz, CA, U.S.A.) and anti-GAPDH antibodies (Abcam, Cambridge, MA, U.S.A.) overnight at 4°C. After washing five times (6 min each time), the membranes were incubated with horseradish peroxidase-conjugated secondary antibodies (Abcam) for 1 h at room temperature. The bands were visualized by using an enhanced chemiluminescent kit (Thermo Fisher Scientific). Immunopositive bands were detected using a FluorChem M system (ProteinSimple, San Jose, CA, U.S.A.).

### Proliferation assay

The cells were cultured 1000 cells per well. The cell proliferation rates were recorded every 24 h using CCK8 assay (Beyotime, C0012, Shanghai, China).

### Cell survival rates

The rate of cell death was measured using trypan blue (C0011; Beyotime, Shanghai, China) staining. The cells were digested for 3 min and then pipetted thoroughly.

### Transwell assay

Resuspended cells with 100 μl serum-free medium were plated in 12-well transwell chamber of each insert (8 μm pore size, Corning, U.S.A.) with a Matrigel coated membrane (BD Bioscience, San Jose, U.S.A.) for the transwell invasion assay. Lower chambers of the inserts were filled with DMEM medium with 10% FBS. Chambers were incubated at 37°C in a humidifed incubator containing 5% CO_2_. About 24 h later, cells invaded to the lower surface of the insert were fixed, stained, and counted under a light microscope.

### Nuclear and chromatin RNA fraction

Nuclear and cytoplasmic fractions of HCT-116 cells were partitioned using a PARIS Kit (Thermo Fisher Scientific, Rockford, IL, U.S.A.). A total of 1.0 × 10^7^ cultured cells were collected, placed on ice, and resuspended with 500 µl ice-cold cell fractionation buffer. All the samples were centrifuged at 500 ***g*** for 5 min, and the cytoplasmic fraction was carefully aspirated from the nuclear pellets fraction. Washing the pellet twice in ice-cold cell fractionation buffer to prevent contamination of the nuclear fraction with components from the cytoplasmic fraction.

### Organ collection

Six C57/B6 mice were killed and the brain, heart, liver, spleen, lung, kidney, and pancreas were collected and using the TRIzol reagent (Thermo Fisher Scientific). The total RNA was extracted as we showed described below.

### Statistical analysis

All the graphs-plotting and data analysis were performed by using the SPSS software 21.0. All the data were shown as mean ± S.D. The significant differences between different groups were analyzed by t-test (comparison for two groups), or X^2^-testor one-way ANOVA (comparison for more than two groups). *P*<0.05 was considered to be statistically significant.

## Results

### Bioinformatics analysis of lncRNA FAM3D-AS1

**Figure 1:**
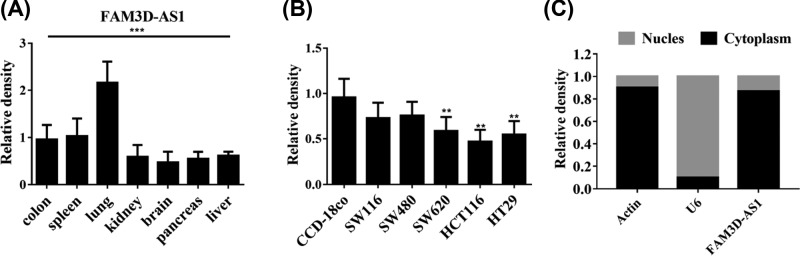
Bioinformatics analysis of lncRNA FAM3D-AS1 (**A**) Multitissue assay revealed that lncRNA FAM3D-AS1 mainly expressed in lung tissues. (**B**) lncRNA FAM3D-AS1 was significantly reduced in CRC cell lines. (**C**) lncRNA FAM3D-AS1 mainly located in cytoplasm. **P<0.01, ***P<0.001.

To investigate the function of FAM3D-AS1, we first performed bioinformatics analysis and explored the biological features of FAM3D-AS1. FAM3D-AS1 can be detected in multiple tissues using real-time PCR. To our surprise, the expression of FAM3D-AS1 was highly in lung tissue, suggesting that FAM3D-AS1 may also possess function in lung (Figure 1A). Next, we verified the expression of FAM3D-AS1 in different colon cells. The expression of FAM3D-AS1 was significantly decreased in colorectal cells (Figure 1B). We also performed cyto-nucles separation assay to identify the location of FAM3D-AS1, which was mainly located in cytoplasm (Figure 1C). These results suggested that FAM3D-AS1 may possess function in CRC.

### Functional analysis of lncRNA FAM3D-AS1

**Figure 2:**
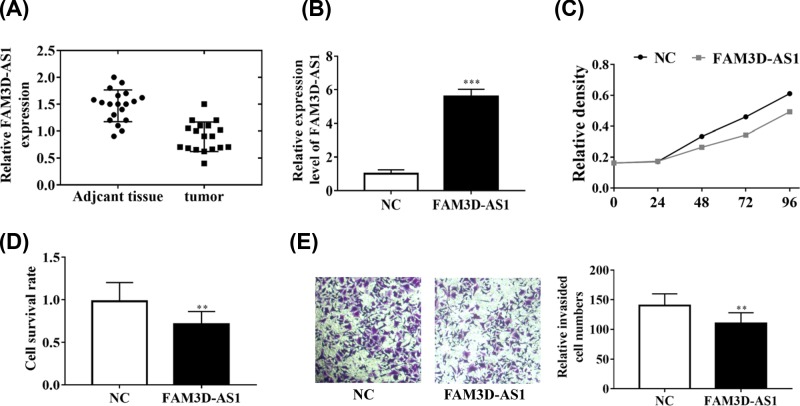
Functional analysis of lncRNA FAM3D-AS1 (**A**) The expression of lncRNA FAM3D-AS1 was reduced in CRC tissues. (**B**) The expression of lncRNA FAM3D-AS1 was significantly increased about six times fold. (**C**) Overexpression of FAM3D-AS1 inhibited the cell proliferation. (**D**) The cell survival rates were reduced in FAM3D-AS1 overexpressed group. (**E**) Overexpression of FAM3D-AS1 inhibited cell invasion. **P<0.01, ***P<0.001.

To explore the function of FAM3D-AS1 in CRC, we first detected the expression of FAM3D-AS1 in CRC. The expression of FAM3D-AS1 was manifestly reduced in CRC compared with adjacent tissues (Figure 2A). Next, we constructed the overexpressed vector. The expression of FAM3D-AS1 was manifestly increased about six times fold in HCT116 cells (Figure 2B). Cell proliferation, death rates, and invasion were performed to evaluate the function of FAM3D-AS1. First, CCK8 assay was performed and we found that overexpression of FAM3D-AS1 significantly inhibited the cell proliferation compared with the NC group (Figure 2C). Moreover, overexpression of FAM3D-AS1 also promoted the cell death rates (Figure 2D). The invasided cell numbers were reduced after the overexpression of FAM3D-AS1, suggesting that FAM3D-AS1 can inhibit the cell invasion (Figure 2E), Thus, these results revealed that FAM3D-AS1 possessed the function of inhibition of cell proliferation and invasion.

### Knockdown of lncRNA FAM3D-AS1 promoted cell proliferation and invasion

**Figure 3:**
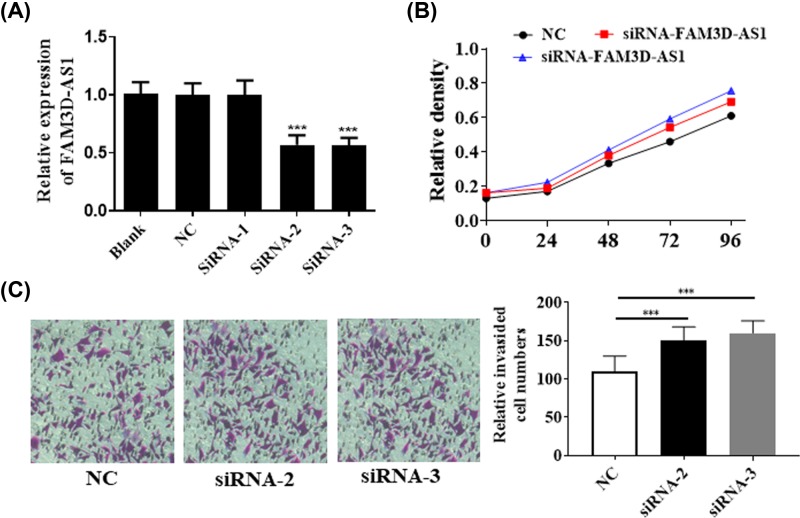
Knockdown of lncRNA FAM3D-AS1 promoted cell proliferation and invasion (**A**) Knockdown of lncRNA FAM3D-AS1 was verified via qPCR. (**B**) Knockdown of lncRNA FAM3D-AS1 significantly promoted cell proliferation. (**C**) Knockdown of lncRNA FAM3D-AS1 significantly promoted cell invasion. ***P<0.001.

To further investigate the function of lncRNA FAM3D-AS1 in CRC, we knockdown the expression of lncRNA FAM3D-AS1 via siRNA. As shown in Figure 3A, the expression of lncRNA FAM3D-AS1 was reduced in siRNA-2 and siRNA-3. Knockdown of lncRNA using siRNAs significantly promoted cell proliferation via CCK-8 assay (Figure 3B). In addition, we also performed transwell assay to analyze the invasion effect. Our results showed that lncRNA FAM3D-AS1 significantly prompted the cell invasion (Figure 3C). Thus, the results demonstrated that knockdown of lncRNA FAM3D-AS1 promoted the cell proliferation and invasion.

### Overexpression of lncRNA FAM3D-AS1 reversed the EMT process

**Figure 4:**
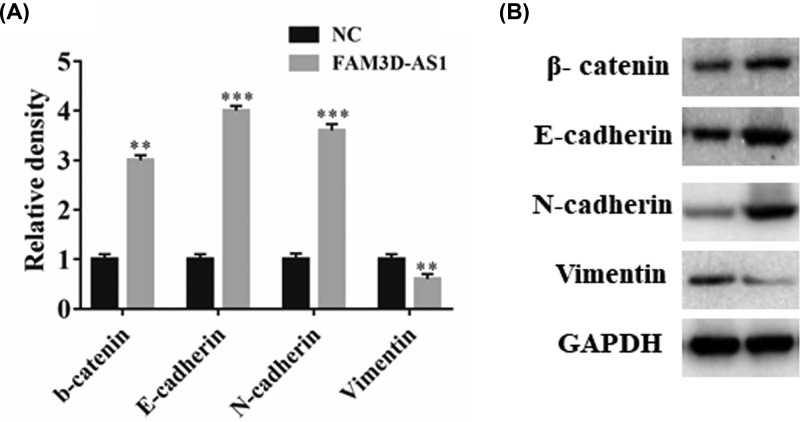
Overexpression of lncRNA FAM3D-AS1 reversed the EMT process (**A**) The EMT related genes was detected using real-time PCR. (**B**) The EMT related genes was detected using western blotting. **P<0.01, ***P<0.001

It is known that cell invasion and EMT process is a key mechanism that associated with cell metastasis. Therefore, we try to assess the EMT related markers. First, real-time PCR was performed to detect the gene expression. The EMT related genes, β-catenin, E-cadherin, and N-cadherin, were significantly reduced compared with NC groups, whereas vimentin was slightly reduced (Figure 4A). We further performed western blotting to detect the protein level of these markers. Similar results were observed in FAM3D-AS1 overexpressed group (Figure 4B). Thus, these results suggested that FAM3D-AS1 reversed the EMT process.

### FAM3D-AS1 may function through NF-κB signaling pathway

**Figure 5:**
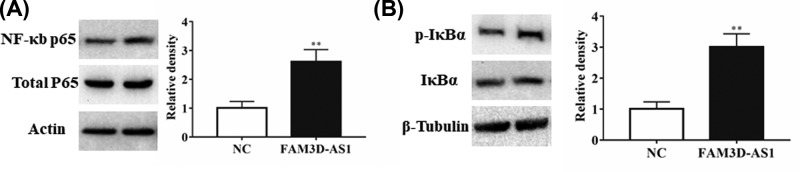
FAM3D-AS1 may function through NF-κB signaling pathway (**A**) Overexpression of FAM3D-AS1 influenced the p65 expression. (**B**) FAM3D-AS1 promoted the level of phosphorylated IκBα. **P<0.01.

NF-κB signaling pathway has been proved that play an important role in CRC [19–22]. Since our previous results have shown that FAM3D-AS1 can influence the EMT process, we detected whether FAM3D-AS1 could influence the NF-κB signaling pathway. We analyzed the levels of p65, a major subunit of NF-κB, which was significantly increased in the FAM3D-AS1 overexpressed group compared with normal control (Figure 5A). Phosphorylation-dependent degradation of IκBα in the cytosol is required for nuclear translocation of NF-κB. We found that FAM3D-AS1 promoted the level of phosphorylated IκBα (Figure 5B). These data indicated that FAM3D-AS1 may function through NF-κB signaling pathway.

## Discussion

Although numerous differential lncRNAs have been identified, the relationship between lncRNA and CRC still remains largely unclear. In the present study, we investigated the function and provided a possible mechanism of lncRNA FAM3D-AS1 in CRC. We found that lncRNA FAM3D-AS1 located in cytoplasm. Overexpression of FAM3D-AS1 significantly inhibited the cell proliferation, cell survival rates and invaded cells. We also provided evidence that FAM3D-AS1 reversed the EMT process. Moreover, we proved that FAM3D-AS1 inhibits CRC development through NF-κB signaling pathway. Taken together, we performed functional analysis of FAM3D-AS1 and provided a possible mechanism in the development of CRC. Our study provided new targets for clinical treatment.

Recently, numerous studies have identified the role of lncRNA in CRC. For example, high expression of lncRNA MALAT1 suggests a biomarker of poor prognosis in CRC [23]. A lncRNA, lncRNA-ATB, is involved in the progression and prognosis of CRC [24]. Besides, many researchers have paid their attention to the study of role of lncRNA in different physiological and pathological process. Increased expression of lncRNA BANCR is associated with clinical progression and poor prognosis in gastric cancer [25]. Thus, exploring new lncRNA will provide more evidences to understand the complicated biological process of CRC. In our study, we proved that lncRNA FAM3D-AS1 inhibited invasion, proliferation, and EMT through regulating NF-κB signaling pathway.

EMT is characterized by loss of intercellular contacts and increasing components of the contractile cytoskeleton [26]. Numerous studies have demonstrated that EMT plays significant role in the process of invasion and metastasis of tumor development [27]. In our study, we found that β-catenin, E-cadherin, and N-cadherin were significantly reduced compared with NC groups, whereas vimentin was slightly reduced, suggesting that FAM3D-AS1 can reverse the EMT process.

Although we provided the function and role of lncRNA FAM3D-AS1 in CRC, its direct downstream regulator still remains unknown. There still exists some problems that need clarification. For example, whether lncRNA FAM3D-AS1 exist different isoforms but still remains unknown and northern blot experiments are needed to verify this. The mechanism of FAM3D-AS1 inhibited the progress and development of CRC is clearly warranted.

In summary, we explore the function and mechanism of lncRNA FAM3D-AS1 in CRC. We found that lncRNA FAM3D-AS1 inhibited the cell proliferation, invasion, and EMT process through NF-κB signaling pathway. Our study provided new insights and treatment target for CRC.

## Abbreviations

ATCC: American Type Culture Collection
CRC: colorectal cancer
IncRNA: long noncoding RNA
qRT-PCR: quantitative real-time PCR
