# Expression of Concern: Long non coding RNA FAM3D-AS1 inhibits development of colorectal cancer through NF-kB signaling pathway

**DOI:** 10.1042/BSR-20190724_EOC

**Published:** 2021-04-07

**Keywords:** Colorectal cancer, EMT, lncRNA

The Editorial Office has been made aware of potential issues surrounding the scientific validity of this paper, hence has issued an expression of concern to notify readers whilst the Editorial Office investigates.

